# Host ABCE1 is at Plasma Membrane HIV Assembly Sites and Its Dissociation from Gag is Linked to Subsequent Events of Virus Production

**DOI:** 10.1111/j.1600-0854.2006.00524.x

**Published:** 2007-01-15

**Authors:** Julia E Dooher, Bobbie L Schneider, Jonathan C Reed, Jaisri R Lingappa

**Affiliations:** 1 Department of Pathobiology, University of Washington 1959 NE Pacific Street, Seattle, WA 98195, USA; 2 Fred Hutchinson Cancer Center 1100 Fairview Avenue, Seattle, WA 98109, USA; 3 Program in Molecular and Cellular Biology, University of Washington 1959 NE Pacific Street, Seattle, WA 98195, USA; 4 Department of Medicine, University of Washington 1959 NE Pacific Street, Seattle, WA 98195, USA

**Keywords:** ABCE1, assembly, capsid, EM, Gag, HIV, HP68, immunogold, plasma membrane, pulse-chase, RLI

## Abstract

In primate cells, assembly of a single HIV-1 capsid involves multimerization of thousands of Gag polypeptides, typically at the plasma membrane. Although studies support a model in which HIV-1 assembly proceeds through complexes containing Gag and the cellular adenosine triphosphatase ABCE1 (also termed HP68 or ribonuclease L inhibitor), whether these complexes constitute true assembly intermediates remains controversial. Here we demonstrate by pulse labeling in primate cells that a population of Gag associates with endogenous ABCE1 within minutes of translation. In the next ∼2 h, Gag–ABCE1 complexes increase in size to approximately that of immature capsids. Dissociation of ABCE1 from Gag correlates closely with Gag processing during virion maturation and occurs much less efficiently when the HIV-1 protease is inactivated. Finally, quantitative double-label immunogold electron microscopy reveals that ABCE1 is recruited to sites of assembling wild-type Gag at the plasma membrane but not to sites of an assembly-defective Gag mutant at the plasma membrane. Together these findings demonstrate that a population of Gag present at plasma membrane sites of assembly associates with ABCE1 throughout capsid formation until the onset of virus maturation, which is then followed by virus release. Moreover, the data suggest a linkage between Gag–ABCE1 dissociation and subsequent events of virion production.

HIV-1 Gag polypeptides are synthesized and myristoylated in the cytoplasm of infected cells, and then subsequently traffic to the cytoplasmic face of the plasma membrane or other membranes for capsid formation. Assembly of a single capsid involves multimerization of ∼5000 Gag polypeptides (1), as well as encapsidation of two copies of HIV-1 genomic RNA and other viral proteins. Immature capsid assembly is followed by capsid maturation, which is mediated by the HIV-1 protease, as well as by budding and virus release. Although Gag has self-assembling properties (2–4), the complexity of assembly in cells raises the possibility that cellular proteins may play critical roles during Gag assembly. A number of studies support a model of HIV-1 capsid assembly in which Gag assembles into capsids by progressing through a post-translational pathway of assembly intermediates, requiring involvement of the cellular adenosine triphosphatase (ATPase) ABCE1 (5–9). Initial evidence for this model was acquired through studies in a cell-free system that reconstitutes immature HIV-1 capsid formation in a eukaryotic cell extract. Pulse-chase studies in this system initially revealed the existence of assembly intermediates by demonstrating that HIV-1 Gag progresses sequentially through a series of high-molecular-weight complexes during capsid assembly. Cell-free studies also demonstrated that energy is required post-translationally at a specific point in this pathway for completion of assembly (8). The observation that ATP is utilized during assembly led to the identification of an ATP-binding cellular protein, ABCE1 [previously termed HP68 or ribonuclease (RNAse) L inhibitor], which associates with assembling Gag polypeptides in high-molecular-weight assembly intermediates. Studies using depletion–reconstitution in the cell-free system demonstrated that ABCE1 is critical for post-translational events of capsid assembly and have been confirmed using a dominant-negative mutant in primate cells (9).

Although HIV-1 capsid assembly intermediates were first identified in a cell-free HIV-1 capsid assembly system, subsequent studies of primate cells expressing HIV-1 or other primate lentiviral Gag proteins identified ABCE1-containing complexes that correspond to assembly intermediates in their size, composition and energy sensitivity (5,9). However, numerous questions remain unanswered about these intracellular complexes. Here we use pulse-chase radiolabeling in primate cells to demonstrate that Gag is associated with ABCE1, from Gag synthesis until completion of Gag assembly and the onset of Gag polypeptide processing during capsid maturation. A proviral mutant containing an inactivated protease results in delayed release of virus, as expected from studies of others (10), but also prolongs the association of Gag with ABCE1. These data raise the possibility that dissociation of ABCE1 from Gag at the completion of assembly is linked to subsequent events of protease maturation. Moreover, while Gag is associated with ABCE1, it increases in size from relatively small complexes to large complexes that approach the size of completed immature capsids. Finally, we demonstrate via quantitative immunogold electron microscopy (EM) double labeling that wild-type Gag recruits ABCE1 to assembling structures at the plasma membrane, while an assembly-defective Gag mutant that targets to the plasma membrane does not recruit this cellular protein. Notably, using this ultrastructural approach, we demonstrate that electron-dense structures at the plasma membrane that have long been recognized as assembling virions appear to constitute assembly intermediates containing Gag and ABCE1.

## Results

### Kinetic analysis reveals that disappearance of intracellular Gag–ABCE1 complexes correlates with appearance of p24Gag in the medium

**Figure 1:**
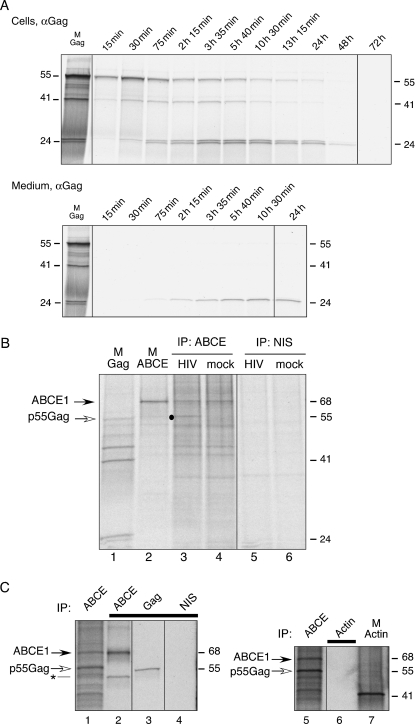
Sequential immunoprecipitation validates the finding that radiolabeled p55Gag associates with ABCE1 A) COS-1 cells expressing HIV-1 were pulsed for 6 min with ^35^S-methionine/cysteine and chased with unlabeled medium for 15 min to 72 h as indicated. Cell lysates (A, top panel) and medium (A, bottom panel) were harvested, denatured, and subjected to immunoprecipitation with αGag. Marker lane (M Gag) indicates the position of full-length Gag and Gag cleavage products. B) COS-1 cells transfected with HIV (lanes 3 and 5) or mock plasmid (lanes 4 and 6) were pulsed for 45 min with ^35^S-methionine/cysteine and chased with unlabeled medium for 15 min. Lysates were subjected to immunoprecipitation under native conditions with αABCE1 (IP: ABCE) or nonimmune serum (IP: NIS). Lanes 1 and 2 are marker lanes (M) that indicate the position of Gag and ABCE1. Dot indicates position of coimmunoprecipitated p55Gag in lane 3. C) COS-1 cells expressing HIV were pulsed for 1 h with ^35^S-methionine/cysteine and chased with unlabeled medium for 15 min. Lysates were subjected to native immunoprecipitation with αABCE1 (ABCE, lanes 1 and 5), and eluates from these initial immunoprecipitations were programmed into a second round of immunoprecipitations, indicated with black bar, using αABCE1 (lane 2), αGag (lane 3), nonimmune antibody (lane 4) or actin antibody (lane 6). Marker indicates position of actin (M actin, lane 7). Asterisk indicates position in lane 2 of a frequently seen ∼45-kD band that cross-reacts with αABCE1. Molecular weight markers or positions of ABCE1 or Gag are indicated beside all panels. All lanes in a given panel are from the same autoradiograph, with dividing lines indicating where lanes from other regions of the same autoradiograph are spliced. All data are representative of four independent experiments.

Previously, using a cell-free HIV-1 capsid assembly system, we demonstrated that ABCE1 associates only transiently with p55Gag, releasing from p55Gag upon completion of capsid assembly (9). Consistent with this, ABCE1 is not found in virions released from cells but is associated with intracellular p55Gag at steady state (5). If complexes containing HIV-1 p55Gag and ABCE1 in primate cells represent transient post-translational assembly intermediates as predicted by results obtained in the cell-free capsid assembly system, then a kinetic analysis in cells should reveal that these complexes appear shortly after completion of p55Gag synthesis and disappear as assembly is completed and virions are released. To analyze the kinetics of p55Gag progression through these ABCE1-containing complexes, we pulse-labeled cells and used immunoprecipitation to follow these radiolabeled complexes over time. Before examining complexes containing p55Gag and ABCE1, we first determined the time-course of p55Gag synthesis and release into the medium by immunoprecipitating cell lysates and medium with antibody to Gag (αGag) after denaturation. COS-1 cells expressing an HIV-1 provirus with the viral *env* gene deleted (HIV) were pulse labeled for 6 min with ^35^S-methionine/cysteine and chased for different periods of time with unlabeled medium. Synthesis of radiolabeled p55Gag peaked at 30 min into the chase and then decreased until p55Gag was no longer visible at >24 h into the chase period (Figure 1A, Cells). Over time, p55Gag was cleaved into 41-, 25- and 24-kD polypeptides that were immunoprecipitated by αGag (directed against the p24 capsid domain of Gag), consistent with processing of the p55Gag precursor protein by the HIV-1 protease during the virus maturation step that follows assembly. Gag began to appear in the medium in the form of p24Gag at 75 min into the chase period and continued to accumulate in the medium for over 24 h (Figure 1A, Medium).

Next, we examined the formation of complexes containing p55Gag and ABCE1 in radiolabeled cells. Previously, we used immunoprecipitation with affinity-purified antibody to ABCE1 (αABCE1) followed by Western blotting with αGag to show that p55Gag associates with endogenous unlabeled ABCE1 in primate cells at steady state (5,9). To determine whether newly synthesized p55Gag in radiolabeled HIV-expressing cells associates with endogenous ABCE1, we performed immunoprecipitations under native conditions (which preserve protein–protein interactions) on cell lysates from radiolabeled COS-1 cells transfected with HIV or mock plasmid. We observed immunoprecipitation of a prominent 68-kD radiolabeled protein in both HIV-expressing and mock cells that aligned with an ABCE1 marker (Figure 1B, compare lanes 3 and 4 to 2). In addition, a prominent 55-kD radiolabeled band that aligned with a Gag marker (Figure 1B, compare lanes 3 to 1) was coimmunoprecipitated by αABCE1 from HIV-expressing cells, but not from mock-transfected cells (Figure 1B, compare lanes 3 and 4). Immunoprecipitations using nonimmune serum revealed that immunoprecipitated bands were specific to αABCE1 (Figure 1B, lanes 5 and 6). It should be noted that a number of other radiolabeled bands were also coimmunoprecipitated by αABCE1 under native conditions in both mock and HIV-expressing cells (Figure 1B, lanes 3 and 4). The pattern of radiolabeled bands coimmunoprecipitating with αABCE1 appeared identical in immunoprecipitations from HIV and mock-transfected samples, with the exception of the 55-kD band that was only immunoprecipitated from cells expressing HIV. ABCE1, which is critical for ribosome biogenesis, nuclear export of ribosomes and translation initiation (11–14), binds to translation initiation factors, such as eIF2 (11),(12), eIF3 (11,12,14) and eIF5 (11,12), as well as a variety of ribosomal proteins (13). Thus, it is likely that the other proteins coimmunoprecipitated by αABCE1 represent these and other factors, most likely in multiple separate complexes.

To ensure that the 55-kD protein represents HIV-1 Gag and not another protein of similar size, we performed serial immunoprecipitations on eluates from αABCE1 immunoprecipitations. COS-1 cells expressing HIV were subjected to immunoprecipitation with αABCE1 under native conditions, followed by elution in 1% SDS (Figure 1C, lanes 1 and 5). The denatured eluate was diluted, divided into equivalent aliquots and subjected to immunoprecipitation using antibodies to ABCE1, Gag, actin or nonimmune serum. This second round of immunoprecipitations confirmed that radiolabeled ABCE1 and p55Gag were present in the eluate of the original αABCE1 immunoprecipitation (Figure 1C, lanes 2 and 3, respectively), while actin was absent (Figure 1C, lane 6), and the nonimmune control revealed no bands (Figure 1C, lane 4). Gag was also immunoprecipitated from the eluate of the αABCE1 immunoprecipitation using two other antibodies directed against Gag (J.E. Dooher and J.R. Lingappa, unpublished data). These results confirm that newly synthesized full-length Gag associates with endogenous ABCE1 in radiolabeled cells, as expected.

**Figure 2:**
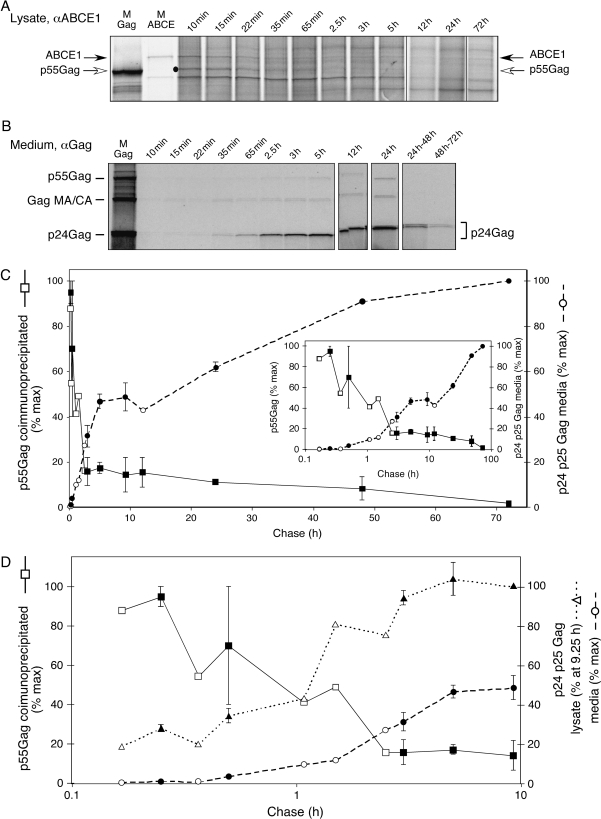
Intracellular complexes containing radiolabeled Gag and ABCE1 appear shortly after translation and disappear upon intracellular processing of radiolabeled p55Gag A) COS-1 cells expressing HIV-1 were pulsed for 15 min with ^35^S-methionine/cysteine and chased with unlabeled media. At chase times indicated above panels, cells were harvested and lysates were subjected to immunoprecipitation under native conditions with αABCE1. Dot indicates position of coimmunoprecipitated p55Gag. B) Collections of medium at chase times indicated were subjected to immunoprecipitation with αGag. Medium collections in the first 24 h were cumulative, starting with the beginning of the chase period, while medium collected at later times shows p24Gag release during intervals (24–48 h and 48–72 h). Marker lanes (M Gag, M ABCE) show the positions of p55Gag and ABCE1. All lanes are from the same autoradiograph, with lanes spliced from elsewhere on the same autoradiograph indicated by dividing line. C) The amount of radiolabeled p55Gag coimmunoprecipitated with αABCE1 in A was quantitated as percentage of maximum (squares, left-hand y-axis) and was plotted against the amount of radiolabeled Gag (p24Gag and p25Gag) present in medium in B as percentage of maximum radiolabeled p24Gag and p25Gag accumulated at 72-h chase (circles, right-hand y-axis). Inset shows data with chase time plotted on a logarithmic scale in order to highlight early time-points. D) Data shown in C are plotted against increase in processed Gag bands (p24Gag and p25Gag) from cell lysates (triangles, right-hand y-axis) and against the amount of radiolabeled Gag (p24Gag and p25Gag) present in medium as in C (circles, right-hand y-axis). Graph shows data with chase time plotted on a logarithmic scale in order to highlight early time-points (up to 9.25-h chase). Accumulation of processed p24Gag and p25Gag bands in lysate (triangles, right-hand y-axis) was quantitated as percentage p24Gag and p25Gag present at maximum intracellular levels (9.25 h). Processed Gag bands in the media were quantitated as percentage of maximum radiolabeled p24Gag and p25Gag accumulated at 72-h chase (circles, right-hand y-axis). C,D) When identical time-points were available in two experiments (filled squares, circles or triangles), data were averaged, with error bars showing SEM. Open squares, circles and triangles show time-points that were only examined in one of the two experiments. All data are representative of four independent experiments.

Next, we examined the time-course of Gag association with ABCE1 by radiolabeling COS-1 cells expressing HIV for 15 min and performing αABCE1 coimmunoprecipitations under native conditions at different chase times. p55Gag was clearly associated with ABCE1 at the 10 and 15 min chase times and was associated to a lesser extent at 22–65 min (Figure 2A, black dot). Minimal association of Gag with ABCE1 was observed from 65 min to 5 h, and after that, no significant association was detected. Similar results were obtained when lysates were harvested in 10 mm ethylenediaminetetraacetic acid (EDTA) or in RNAse A (Figure S1), indicating that this association was not dependent on intact ribosomes or polysomes. Thus, a population of newly synthesized p55Gag appeared to be associated with ABCE1 in the first ∼5 h of the chase period. Next, we compared the kinetics of complexes containing p55Gag and ABCE1 with the appearance of the p24Gag cleavage product in the medium (representing released, mature virus). Medium collected at different chase times was denatured and subjected to immunoprecipitation with αGag. Autoradiographs demonstrated that radiolabeled p24Gag began appearing in the medium at 35–65 min (Figure 2B).

To compare the kinetics of the Gag–ABCE1 complex to the kinetics of Gag release, the amount of radiolabeled p55Gag associated with ABCE1 in cell lysates and the amount of radiolabeled p24Gag present in the medium were quantified and graphed. Radiolabeled Gag appeared to be released into the medium in two phases. In the first phase, which lasts for ∼ 5 h after pulse labeling, radiolabeled p24Gag was released at a rapid rate, while subsequent release occurred at a slower rate (Figure 2C). These graphs also revealed that the disappearance of intracellular Gag–ABCE1 complexes during the first 5 h into the chase period was inversely correlated with the appearance of p24Gag in the medium. The loss of Gag–ABCE1 complexes at this stage is consistent with dissociation of ABCE1 from Gag upon completion of capsid assembly, which we had documented previously in a cell-free system for HIV-1 capsid assembly [figure S1 in Zimmerman et al. (9)]. In the cell-free system, total radiolabeled Gag and radiolabeled Gag–ABCE1 complexes could be accurately quantified, thereby ruling out degradation and confirming ABCE1 dissociation as the reason for the loss of Gag–ABCE1 complexes at the completion of assembly. To date, we have observed an excellent correspondence between results obtained in the cell-free system for HIV-1 capsid assembly and results obtained in cells (5,7–9), but it remains possible that the loss of intracellular Gag–ABCE1 complexes is due to their degradation rather than due to dissociation of ABCE1 from Gag.

Notably, a delay was seen when comparing the loss of intracellular Gag–ABCE1 complexes to the appearance of virus in the medium (Fig. 2c). For example, 50% of Gag–ABCE1 complexes were lost by 2 h into the chase, while ∼10 h elapse before 50% of virus was released into the medium. This apparent lag between the likely dissociation of ABCE1 from p55Gag and appearance of p24Gag in the medium could be explained by the time it takes for proteolytic processing and/or budding, which follow completion of immature capsid formation. When we compared the disappearance of Gag–ABCE1 complexes to the appearance of intracellular p24Gag and p25Gag in these experiments (Figure 2D), we observed very close timing without a delay. Thus, loss of the Gag–ABCE1 association appeared to correlate very closely in time with the onset of intracellular processing of p55Gag, which was followed, after a delay, by virus release. Together, these data indicate that a population of p55Gag is associated with ABCE1 from the time of synthesis until the onset of p55Gag processing.

### An assembly-incompetent Gag mutant fails to form ABCE1 complexes, whereas an assembly-competent HIV mutant displays prolonged Gag association with ABCE1

**Figure 3:**
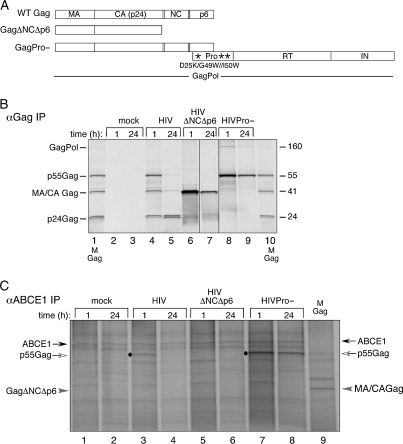
Wild-type HIV-1 Gag associates with ABCE1 transiently, while Gag from HIVΔNCΔp6 fails to associate with ABCE1, and Gag from HIVPro− shows prolonged association with ABCE1 A) Schematic of HIV constructs used. Gag encodes matrix (MA), capsid (CA), nucleocapsid (NC) and p6, while GagPol also encodes the HIV enzymes protease (Pro), reverse transcriptase (RT) and integrase (IN). Asterisks indicate sites of point mutations in HIVPro− construct. B,C) COS-1 cells transfected with the indicated constructs were pulsed for 1 h with ^35^S-methionine/cysteine and chased with unlabeled media. Lysates were harvested at 1 or 24 h into the chase period (as indicated) and were immunoprecipitated with αGag after denaturation (B) or with αABCE1 under native conditions (C). Markers for GagPol, p55Gag and Gag cleavage products (MA/CA Gag, p24Gag) are shown (M Gag), and positions of ABCE1, p55Gag and GagΔNCΔp6 are indicated by black, white and gray arrows, respectively in (C). Dots show the position of coimmunoprecipitated p55Gag in lanes 3, 7 and 8. All lanes in a given panel are from the same autoradiograph, with dividing lines indicating where lanes from other regions of the same autoradiograph are spliced. Data are representative of three independent experiments.

Previously, we demonstrated that an assembly-defective Gag mutant truncated proximal to the nucleocapsid (NC) domain (GagΔNCΔp6; Figure 3A) is not coimmunoprecipitated by αABCE1 under steady-state conditions (5,8,9,15). Furthermore, a mutational analysis demonstrated that residues in NC, but not p6, are critical for the Gag–ABCE1 association (7). To confirm that an assembly-defective Gag mutant fails to bind ABCE1 in a kinetic assay, as is the case in a steady-state context, COS-1 cells expressing HIV or HIV-encoding GagΔNCΔp6 (HIVΔNCΔp6, HIV-1 provirus with a deletion in *env* and a truncation in Gag proximal to NC) and mock-transfected cells were pulsed for 1 h and subjected to immunoprecipitation under denaturing conditions with αGag (Figure 3B) or to immunoprecipitation under native conditions with αABCE1 (Figure 3C) at 1 versus 24 h into the chase period. Immunoprecipitations with αGag demonstrated expression of p55Gag and GagΔNCΔp6 in cells expressing HIV and HIVΔNCΔp6, respectively (Figure 3B). Preparations of virus-like particles (VLPs) demonstrated that cells expressing HIVΔNCΔp6 failed to release virus into the medium, unlike HIV-expressing cells as expected (Figure S2). In cells expressing HIV, p55Gag was associated with ABCE1 at 1 h but not at 24 h (Figure 3C, compare lanes 3 and 4), consistent with previous pulse-chase results (see Figure 2A). In contrast, GagΔNCΔp6 was not associated with ABCE1 at either 1 or 24 h (Figure 3C, at gray arrowhead, lanes 5 and 6), despite excellent expression (Figure 3B, lanes 6 and 7). In fact, the αABCE1 coimmunoprecipitation of HIVΔNCΔp6 looked identical to αABCE1 coimmunoprecipitations from cells transfected with mock plasmid (Figure 3C, compare lanes 5 and 6 to lanes 1 and 2). Thus, pulse labeling demonstrated, as expected, that an assembly-incompetent Gag mutant truncated proximal to NC failed to associate with ABCE1 at a chase time when significant amounts of radiolabeled wild-type Gag were associated with ABCE1.

Because loss of the Gag–ABCE1 association was closely correlated with p55Gag processing (Figure 2D), we also examined the effect of protease inactivation on the Gag–ABCE1 association. In parallel with transfections described above, COS-1 cells were transfected with equivalent amounts of HIVPro−, a proviral construct encoding point mutations that inactivate the HIV-1 protease and cause the release of immature virus rather than mature virus [(16), J.E. Dooher and J.R. Lingappa, unpublished data]. Surprisingly, p55Gag produced by HIVPro− was associated with ABCE1 at both 1 and 24 h (Figure 3C, lanes 7 and 8). Thus, the two HIV-1 mutants we examined have opposite phenotypes, with assembly-defective Gag from HIVΔNCΔp6 failing to form complexes with ABCE1, and p55Gag from the assembly-competent but processing-defective HIVPro− displaying prolonged association with ABCE1.

**Figure 4:**
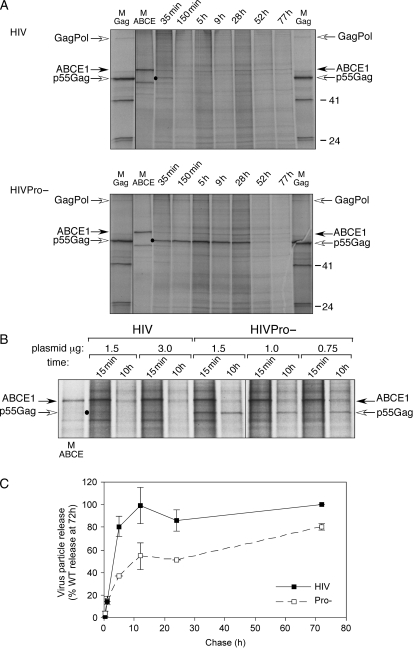
Time-course demonstrates prolonged ABCE1 association with Gag from HIVPro− and reduced rate of HIVPro− particle release A) COS-1 cells expressing 3.0 μg HIV or HIVPro− were radiolabeled for 15 min with ^35^S-methionine/cysteine and chased with unlabeled media. At indicated times, cells were harvested and subjected to immunoprecipitation under native conditions with αABCE1. Markers (M) for the position of Gag and ABCE1 are shown. Dot shows the position of coimmunoprecipitated p55Gag in chase lanes. B) COS-1 cells transfected with indicated amounts of HIV or HIVPro− plasmid (μg) were pulsed for 15 min with ^35^S-methionine/cysteine and chased for 15 min or 10 h with unlabeled media. Lysates were subjected to native immunoprecipitation with αABCE1. Dot shows the position of coimmunoprecipitated p55Gag in chase lanes. Data are representative of three independent experiments. C) VLPs from cells transfected with 1.5 μg wild-type HIV (filled squares) or HIVPro− (open squares) plasmid were prepared using sucrose cushions, and amount of radiolabeled Gag was quantitated and graphed as a percentage of total wild-type virus release. Total wild-type virus release was defined as the amount of wild-type virus accumulated in the medium at 72 h into the chase. Data are averaged from two independent experiments, and error bars indicate SEM. Native immunoprecipitations were performed on cell lysates in C (data not shown) and were similar to 1.5 μg lanes shown in B.

To confirm the prolonged Gag–ABCE1 association in cells expressing HIVPro−, we examined a detailed time-course for HIVPro− versus wild-type HIV from cells that were radiolabeled for 15 min and chased with unlabeled medium. Immunoprecipitations of denatured cell lysate with αGag demonstrated that Gag from HIVPro− failed to be proteolytically cleaved at any time-point (data not shown). When cells expressing wild-type HIV were immunoprecipitated with αABCE1 under native conditions, complexes containing Gag and ABCE1 could be detected for ∼ 2.5 h (Figure 4A). However, in cells expressing HIVPro−, high levels of Gag were associated with ABCE1 for at least 28 h, with reduced association at 52 h, and no detectable association at 77 h (Figure 4A). Notably, the GagPol polyprotein was also coimmunoprecipitated by αABCE1, best seen in the coimmunoprecipitations from cells expressing HIVPro− in Figure 4A. Quantitation revealed that the ratio of radiolabeled Gag to GagPol in the αABCE1 immunoprecipitations of HIVPro− cell lysates was as high as 18:1, with an average from multiple time-points of 10:1, which approximates the Gag to GagPol ratio found in virions (17,18).

To determine whether the prolonged association was simply due to higher intracellular levels of Gag produced by the HIVPro− versus wild-type construct, we transfected parallel plates with different amounts of the HIV and HIVPro− plasmids, labeled them for 15 min and used immunoprecipitation with αABCE1 to examine the association of p55Gag with ABCE1 at 15 min and 10 h into the chase period (Figure 4B). Within the range of plasmid concentrations used, p55Gag from cells expressing wild-type HIV associated with ABCE1 to similar extents at 15 min but was not associated with ABCE1 at 10 h. In contrast, p55Gag expressed from the HIVPro− construct was associated with ABCE1 both at 15 min and at 10 h, even when the amount of the HIVPro− plasmid was reduced to 25% of wild-type plasmid (Figure 4B, compare 0.75 μg HIVPro− to 3.0 μg HIV plasmid). These data indicate that in the absence of p55Gag processing, the half-life of Gag–ABCE1 complexes becomes significantly greater than ∼2 h, unlike when p55Gag is produced from the wild-type HIV construct, and that this effect appears to be independent of p55Gag expression levels. This finding supports the notion that the loss of Gag–ABCE1 complexes at the onset of maturation in cells expressing wild-type HIV-1 likely represents dissociation of ABCE1 from p55Gag rather than degradation of Gag–ABCE1 complexes because mutational inactivation of the HIV-1 protease is unlikely to inhibit cellular degradation processes but could conceivably prolong the interaction of p55Gag with cellular proteins.

Others have demonstrated that protease-defective constructs result in reduced virus release (10). To determine whether the construct exhibiting delayed Gag–ABCE1 dissociation also exhibits reduced virion release, we transfected cells with 1.5 μg wild-type HIV or HIVPro− plasmid, and collected virus particles by sedimenting medium through sucrose cushions at varying times following a 15-min pulse. Aliquots of radiolabeled virion pellets were analyzed directly by SDS-PAGE rather than by αGag immunoprecipitation because p55Gag and p24Gag are immunoprecipitated with different efficiencies by αp24 antibodies and therefore such immunoprecipitations cannot be directly compared. Quantitation revealed that at 5 and 10 h post-transfection, virion release from cells expressing HIVPro− was ∼50% of that released from cells expressing wild-type HIV, but by 72 h the total amount of release between the two groups was fairly similar (Figure 4C). Notably, ABCE1 was not present in immature virions released by HIVPro− constructs (Figure S2), as is the case for wild-type mature HIV-1 particles as well as for immature VLPs produced by expression of simian immunodeficiency virus Gag alone (5). The lack of ABCE1 in released virus is consistent with intracellular dissociation of ABCE1 (Figure 2D). Thus, although protease activity appears to promote efficient dissociation of ABCE1 from p55Gag through a direct or indirect mechanism, it is not required for ABCE1 dissociation from p55Gag. Nevertheless, the finding that inactivation of p55Gag processing correlates with a prolonged half-life of intracellular Gag–ABCE1 complexes and with reduced virion release suggests that dissociation of ABCE1 from Gag is linked to either maturation or virus release.

### Intracellular Gag–ABCE1 complexes undergo a progressive increase in S value

**Figure 5:**
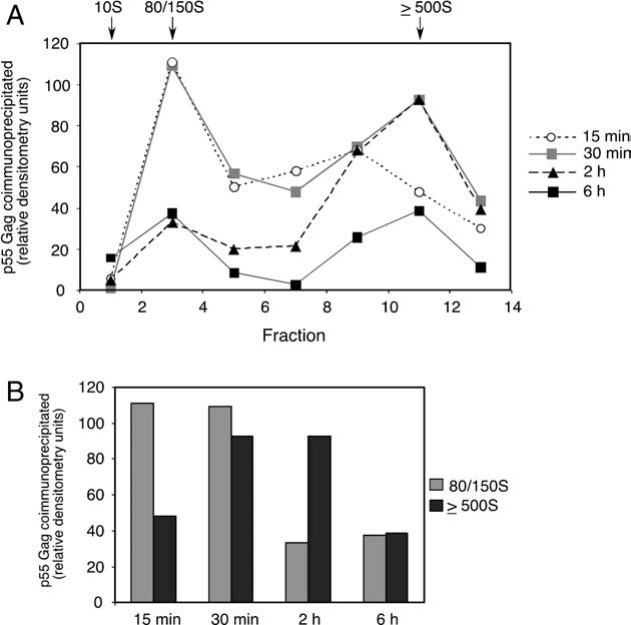
Intracellular complexes containing Gag and ABCE1 increase to approximately the size of capsids over time COS-1 cells expressing HIV-1 were subjected to pulse radiolabeling for 15 min followed by chase with unlabeled medium. At indicated times, cells were harvested and subjected to velocity sedimentation, fractionation and immunoprecipitation under native conditions with αABCE1. A) p55Gag associated with ABCE1 in fractions representing ∼10S ∼80/150S and ≥500S complexes was quantitated and shown as assembly profiles. Positions of ∼10S ∼80/150S and ≥500S complexes are indicated. B) Amount of p55Gag associated with ABCE1 in fractions representing ∼80/150S and ≥500S complexes at indicated time-points was quantitated and graphed. All data are from one experiment and are representative of four independent experiments.

The finding that ABCE1 dissociates from p55Gag at the onset of maturation suggests that ABCE1 is associated with p55Gag throughout the capsid assembly process. To confirm this, we examined whether pulse-labeled Gag–ABCE1 complexes undergo the increase in size over time that would be expected upon multimerization of p55Gag during assembly. Through pulse-chase analysis in a cell-free HIV-1 capsid assembly system, we had previously demonstrated that HIV-1 p55Gag associates with ABCE1 and progresses through discrete assembly intermediates with sedimentation values of ∼80/150S and then ∼500S, culminating in production of the completed ∼750S immature capsid upon dissociation of Gag from ABCE1 (8,9). We had also found that the ∼80/150S intermediate is highly transient in the presence of Mg^2+^, but accumulates when ATP is depleted in the presence of Mg^2+^(5). Consistent with the ATP dependence of the ∼80/150S intermediate, another approach that can be used to trap this complex is to omit Mg^2+^ from harvest and gradient buffers or to add the chelating agent EDTA (Figure S3). For this reason, studies presented here were performed in the absence of Mg^2+^. HIV-expressing COS-1 cells were radiolabeled for 15 min and harvested at various chase times for analysis on velocity sedimentation gradients followed by immunoprecipitation of gradient fractions with αABCE1 under native conditions (Figure 5). At the 15-min chase point, most of the radiolabeled p55Gag associated with ABCE1 was present in complexes that migrated in the ∼80/150S region of the gradient. At 30 min, complexes containing p55Gag and ABCE1 were present to nearly equivalent extents in the ∼80/150S and ∼500S regions of the gradient. At 2 h, there were substantially fewer Gag–ABCE1 complexes in the ∼80/150S region, and the majority of complexes containing p55Gag and ABCE1 were in the form of the ≥500S complex. At this 2-h chase time, the total amount of radiolabeled Gag complexed with ABCE1 had decreased, consistent with significant release of radiolabeled Gag into the medium (see Figure 1A, Medium, and Figure 2B). At 6 h, when much of the radiolabeled Gag had been released into the medium as p24Gag (see Figure 1A, Medium, and Figure 2B), both the ∼80/150S and ≥500S complexes were present in reduced amounts (Figure 5).

It should be noted that radiolabeled amino acids continue to be incorporated into newly synthesized p55Gag until at least 30 min into the chase period (as seen in Figure 1A, Cells). This continued incorporation of radiolabeled amino acids likely reflects the time it takes to deplete pools of radiolabeled aminoacyl transfer RNAs after the labeling medium is removed. Thus, while radiolabeled p55Gag polypeptides that completed synthesis during the first 15 min of the chase shifted from the ∼80/150S complex into the ∼500S complex during the 15- to 30-min chase period, they were replaced by radiolabeled p55Gag polypeptides that were synthesized after the 15-min chase point and formed new ∼80/150S complexes during the 15- to 30-min chase period. This explains why the total amount of radiolabeled ∼80/150S complexes remained relatively constant until after ∼30 min into the chase, when production of newly radiolabeled p55Gag polypeptides ceased (Figure 5B). This also explains why the overall number of Gag–ABCE1 complexes increased until the 30-min chase point. At the 2- and 6-h chase points, when radiolabeled p55Gag was no longer being synthesized, progression of radiolabeled p55Gag from the ∼80/150S complex to the ∼500S complex lead to a decrease in the amount of the ∼80/150S complex, while release of p24Gag into the medium correlated with the progressive decrease in total intracellular Gag–ABCE1 complexes.

Taken together, the pulse-chase and velocity sedimentation analyses strongly support a model in which p55Gag progresses sequentially through intracellular assembly intermediates of increasing size while associated with ABCE1. Upon formation of completed capsids of >500S, ABCE1 dissociates from p55Gag, and p55Gag processing by the HIV-1 protease begins, leading to intracellular capsid maturation followed by budding and release from the cell. Notably, progression of p55Gag over time from ∼80/150S to ∼500S complexes in cells closely corresponds to pulse-chase data from the cell-free HIV-1 capsid assembly system that was originally used to identify HIV-1 capsid assembly intermediates and to define this assembly pathway (8,9).

### Immunogold labeling reveals complexes containing Gag and ABCE1 at the plasma membrane

**Figure 6:**
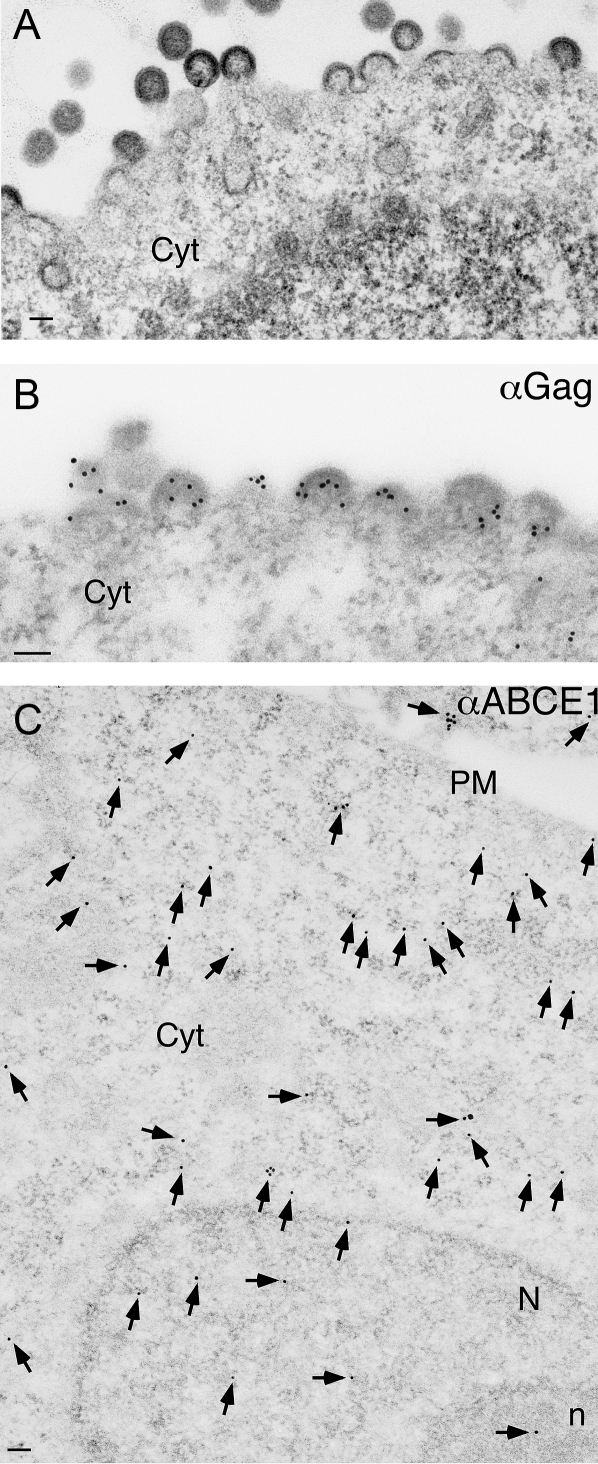
Standard EM and single immunogold labeling of Gag and ABCE1 COS-1 cells transfected with HIV (A,B) or mock plasmid (C) were processed for negative staining transmission EM (A) or for single immunogold labeling with αGag (B; 6-nm gold) or αABCE1 (C; 15-nm gold). In C, all ABCE1 labeling is indicated with arrows. Scale bars correspond to 100 nm. Plasma membrane (PM), cytoplasm (Cyt), nucleus (N) and nucleolus (n) are indicated.

The pulse-chase studies described above demonstrated that ABCE1 associates with assembling p55Gag from the time of p55Gag synthesis, throughout assembly, to the time of capsid maturation and virion release. During this time, the sedimentation value of Gag–ABCE1 complexes increased to approximately that of an immature capsid, most likely because of Gag multimerization. Because the late stages of Gag multimerization are known to occur at membrane sites of assembly, these findings raised the possibility that complexes containing Gag and ABCE1 are located at the plasma membrane. While Gag has been shown to colocalize with endogenous ABCE1 in immunofluorescence studies (9), neither the exact location of Gag–ABCE1 complexes nor the morphologic appearance of capsid assembly intermediates has been described. To address these questions, we examined these complexes ultrastructurally in COS-1 cells expressing HIV. As expected, standard thin-section transmission EM with negative staining revealed the presence of numerous electron-dense structures at the plasma membrane (Figure 6A), which are consistent with assembling and budding HIV-1 capsids observed numerous times by others [reviewed by Wills and Craven (19)]. We next used single immunogold labeling to determine the location of Gag and ABCE1 in cells. For these studies, less fixative and LR White embedding were used to preserve antigenicity necessary for immunogold labeling. Because these conditions result in loss of morphologic detail (20), resolution of fine-structure and electron-dense capsids was reduced. Nevertheless, sites of capsid assembly and budding were still easily recognizable (Figure 6B). Immunogold labeling for Gag alone revealed high-density labeling at sites of assembling and budding capsids at the plasma membrane (Figure 6B), as well as lower density labeling in the cytoplasm of cells expressing HIV. No αGag labeling was seen in mock-transfected cells (J.E. Dooher and J.R. Lingappa, unpublished data).

Immunogold labeling of endogenous ABCE1 in mock-transfected cells revealed labeling throughout the cytoplasm, with minimal labeling at the plasma membrane (Figure 6C). Some ABCE1 labeling was seen in nuclei, especially within nucleoli, consistent with data from other groups indicating that ABCE1 is involved in ribosome export (13,14). Immunogold labeling with either of the gold-conjugated secondary antibodies in the absence of the corresponding primary antibody revealed no labeling (J.E. Dooher and J.R. Lingappa, unpublished data), confirming that all labeling was due to αGag and αABCE1.

### ABCE1 is specifically recruited into sites of HIV-1 assembly at the plasma membrane

**Figure 7:**
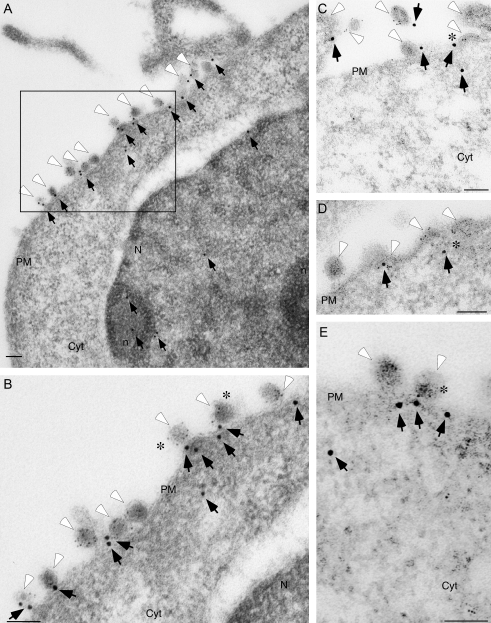
Immunogold labeling of ABCE1 with wild-type Gag reveals colocalization COS-1 cells expressing HIV were processed for immunogold double labeling with αGag (6-nm gold; indicated by white arrowheads) and αABCE1 (15-nm gold; indicated by black arrows). A) Low-power view showing plasma membrane (PM), cytoplasm (Cyt), nucleus (N) and nucleoli (n). B) Magnification of boxed area from A. C–E show high-power views from other fields. Asterisks in B and E show colocalization of Gag and ABCE1 in nearly completed capsids; asterisks in C and D show colocalization of Gag and ABCE1 in partially assembled capsids. Scale bars correspond to 200 nm.

Upon double labeling of Gag (labeled with 6 nm gold) and ABCE1 (labeled with 15 nm gold), low-power electron micrographs of cells expressing HIV revealed high levels of ABCE1 labeling at sites along the plasma membrane (Figure 7A). High-power views of regions with ABCE1 labeling at the plasma membrane indicated colocalization of Gag and ABCE1 at structures representing typical assembling and budding HIV-1 capsids (Figure 7B–E). Colocalization occurred at partially assembled, crescent-shaped capsids that only minimally deformed the plasma membrane (asterisks in Figure 7C,D), as well as at spherical capsids that were nearly completely surrounded by plasma membrane (asterisks in Figure 7B,E).

**Figure 8:**
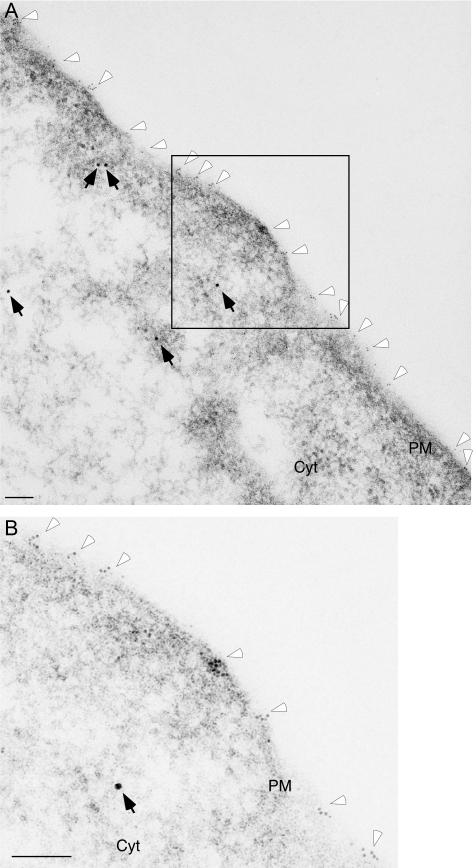
Immunogold labeling of ABCE1 and Gag ΔNCΔp6 demonstrates that they do not colocalize COS-1 cells expressing HIVΔNCΔp6 were processed for immunogold double labeling with αGag (6-nm gold; indicated by white arrowheads) and αABCE1 (15-nm gold; indicated by black arrows). A) Low-power view showing plasma membrane (PM) and cytoplasm (Cyt). B) Inset of boxed area in A. Scale bars correspond to 200 nm.

**Figure 9:**
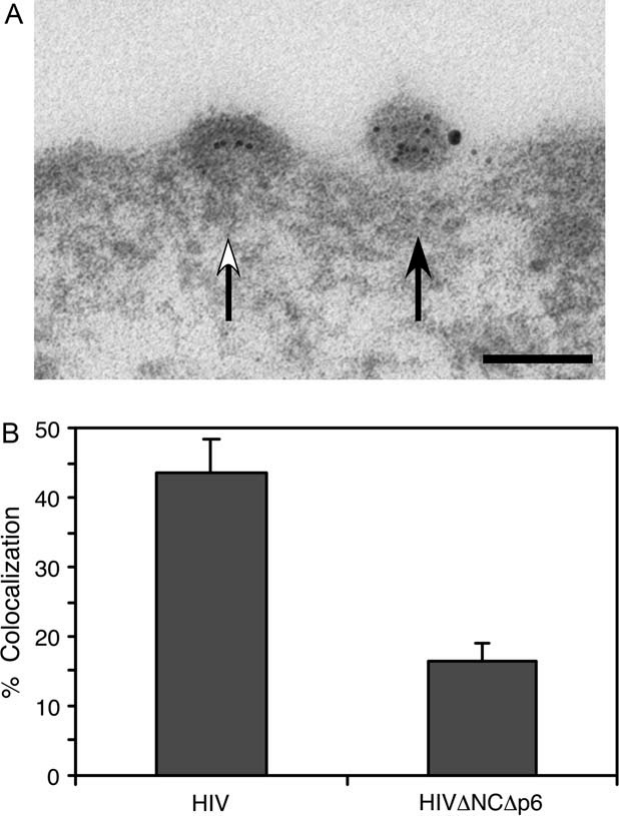
Quantitation of Gag–ABCE1 colocalization at the plasma membrane Sections of COS-1 cells expressing HIV or HIVΔNCΔp6 were analyzed for colocalization of Gag and ABCE1. Colocalization was defined as ABCE1 labeling within 100 nm of clusters of Gag labeling at the plasma membrane. A) Examples of negative (white arrow) and positive (black arrow) colocalization of Gag and ABCE1 are shown. Scale bar corresponds to 100 nm. B) Percentage of Gag clusters that were colocalized with ABCE1 was determined for cells expressing HIV versus HIVΔNCΔp6. Error bars show SEM from three experiments. A total of 64 micrographs of wild-type Gag and 44 micrographs of GagΔNCΔp6, each containing multiple Gag clusters, were counted.

To confirm that ABCE1 specifically colocalizes with assembling Gag, we compared colocalization of ABCE1 with that of wild-type Gag versus the GagΔNCΔp6 mutant, which fails to assemble or associate with ABCE1 (8,9,15) (Figure 3). In cells expressing HIVΔNCΔp6, Gag labeling occurred primarily at the plasma membrane, although, as expected, formation of capsid-like structures was not seen. ABCE1 labeling was diffusely cytoplasmic, with little ABCE1 labeling seen in proximity to Gag labeling at the plasma membrane (Figure 8A,B). This was in complete contrast to the prominent colocalization seen in cells expressing HIV (Figure 7). To assess whether there was a significant difference between these two patterns of colocalization, we quantified the amount of ABCE1 colocalized with wild-type Gag to the amount colocalized with the assembly-defective GagΔNCΔp6 mutant. Colocalization was defined as the presence of ABCE1 labeling within 100 nm of a cluster of Gag labeling, with individual clusters of Gag labeling being at least 100 nm apart (for examples, see Figure 9A). In cells expressing wild-type HIV, ABCE1 was colocalized with ∼ 45% of wild-type Gag clusters present at the plasma membrane (Figure 9B). In contrast, ABCE1 was colocalized with only ∼15% of GagΔNCΔp6 clusters located at the plasma membrane.

To further confirm ABCE1 recruitment into sites of assembly, we performed a separate experiment examining immunogold labeling of ABCE1 and Gag in COS-1 cells expressing either HIVPro− or mock plasmid. Cells expressing HIVPro− were chosen for this comparison to see whether they also revealed recruitment of ABCE1, as was the case for cells expressing wild-type HIV in the previous comparison (Figure 9B). Mock cells were chosen as a control to determine whether the difference in ABCE1 localization was seen when the control lacks Gag expression. Using mock-transfected controls prevented us from directing our analysis to regions of the plasma membrane containing Gag, so instead we examined the density of ABCE1 at virus-producing versus control plasma membrane. From electron micrographs representing six cells expressing HIVPro−, we determined the number of ABCE1 labeling events in 21,568 nm of randomly selected, virus-producing plasma membrane, and compared this to the number of ABCE1 labeling events in 26,678 nm of randomly selected plasma membrane from five mock-transfected cells. In cells expressing HIVPro−, we observed one ABCE1 labeling event per 502 nm of virus-producing plasma membrane, while in the mock cells, we observed one ABCE1 labeling event per 3335 nm of plasma membrane. This indicated that the density of ABCE1 labeling was ∼6.6-fold greater at discrete sites of virus production in cells expressing HIVPro− than at control plasma membranes from mock-transfected cells. Thus, quantitation of ABCE1 labeling in cells expressing either wild-type HIV or HIVPro− indicated that sites of HIV-1 capsid assembly at the plasma membrane contain complexes composed of Gag and ABCE1, and demonstrated specific recruitment of ABCE1 into discrete sites of assembly at the plasma membrane.

## Discussion

Here we used a kinetic analysis to demonstrate that ABCE1, a cellular ATPase, forms intracellular complexes with HIV Gag upon completion of p55Gag synthesis, and appears to dissociate from p55Gag just before capsid maturation and virion release (Figures 2 and 3). Dissociation of ABCE1 from p55Gag appears to be linked to p55Gag polyprotein processing because mutational inactivation of the HIV-1 protease led to prolonged Gag–ABCE1 association (Figure 4). We also found that during the first 2 h after p55Gag synthesis, Gag–ABCE1 complexes undergo a large increase in size that is consistent with immature capsid assembly (Figure 5). Finally, we used an ultrastructural approach (immunogold double labeling; Figures 6–9) to demonstrate that Gag–ABCE1 complexes are present at sites of capsid formation at the plasma membrane. Thus, structures that are the hallmark of HIV-1 assembly in cells and have been documented as sites of assembling p55Gag [e.g. Gelderblom et al. (23), Mergener et al. (24), Schatzl et al. (25)] appear to be assembly intermediates containing p55Gag and at least one cellular factor involved in the assembly process. Together, our biochemical and ultrastructural studies suggest that a population of p55Gag is associated with ABCE1 from the time of p55Gag synthesis, throughout the capsid assembly process, until the onset of capsid maturation, and that this population of Gag is localized to plasma membrane sites of assembly. Moreover, our studies suggest that dissociation of ABCE1 from Gag during capsid assembly is linked to virion maturation or to release by as yet undetermined mechanisms.

Intracellular complexes containing Gag have been reported by many other groups besides ours (26–34). It is likely that many different subsets of Gag complexes exist within a cell, but that only a limited number of these are likely to be intermediates in capsid assembly, with the rest being involved in other late events, or being destined for degradation. Evidence for substantial Gag degradation comes from the finding that when Gag is expressed alone without the rest of the provirus, ∼80% of newly synthesized Gag polypeptides are degraded within 2 h after synthesis (33). In the pulse-chase studies of COS-1 cells expressing genomic HIV or HIVPro− constructs reported here, we also observed that ∼80% of newly synthesized Gag polypeptides fail to be released from cells (J.E. Dooher and J.R. Lingappa, unpublished data), consistent with extensive intracellular Gag degradation. Given such evidence for extensive Gag degradation, we cannot rule out the possibility that intracellular Gag–ABCE1 complexes undergo degradation. Thus, the loss of Gag–ABCE1 complexes during the first 2 h of the chase period in Figure 2D could be due either to dissociation of ABCE1 from Gag at the onset of capsid maturation and virion release or to degradation of Gag–ABCE1 complexes occurring at the same time as processing and release of a different population of Gag polypeptides. However, the very close correlation between the disappearance of Gag–ABCE1 complexes and the onset of intracellular Gag processing in Figure 2D argues in favor of ABCE1 dissociation upon capsid maturation. In addition, results obtained with the mutant containing an inactivated HIV-1 protease (Figure 4) also support the notion that ABCE1 dissociates from p55Gag upon capsid maturation and release. Specifically, when the HIV-1 protease was inactivated, the half-life of Gag–ABCE1 complexes was greatly prolonged (Figure 4), while >80% of total Gag produced in these cells underwent rapid degradation (J.E. Dooher and J.R. Lingappa, unpublished data). It is unlikely that mutational inactivation of Gag polyprotein processing by the HIV-1 would cause a reduction in proteasomal degradation of selected Gag-containing complexes. In light of findings from the cell-free system for HIV-1 capsid assembly documenting that dissociation of ABCE1 occurs upon completion of the immature HIV-1 capsid with total Gag levels remaining constant (9), a more likely explanation is that dissociation of ABCE1 occurs less efficiently when maturation is inhibited and/or virion release is delayed.

Other findings also argue that Gag–ABCE1 complexes constitute the pool of Gag destined for assembly and release rather than a pool of Gag destined for degradation. First, in this study, we demonstrated that during the first 2 h postsynthesis when the vast majority of Gag polypeptides are subject to degradation [(33), J.E. Dooher and J.R. Lingappa, unpublished data], Gag–ABCE1 complexes increase in size, achieving the size of completed immature capsids of >500S, which are much larger than ∼20S proteasomal complexes involved in degradation. Secondly, the finding that Gag–ABCE1 complexes contain p55Gag and GagPol in a ratio of ∼1:10 to 1:20 ratio (Figure 4A), which is characteristic of virions (17,18), also lends support to the notion that these complexes represent nascent virions. Thirdly, we have shown that assembly-incompetent Gag mutants, which likely undergo even greater amounts of degradation than wild-type Gag, fail to form any ABCE1-containing complexes (Figure 3) [also see Lingappa et al. (7), Zimmerman et al. (9), Dooher et al. (35)]. Finally, there are numerous reports of ABCE1 being involved in protein synthesis in many eukaryotic species as described below, but to date, there are no reports of ABCE1 being involved in protein degradation in any species.

The observations that Gag–ABCE1 complexes are at the plasma membrane and that ABCE1 appears to dissociate from Gag upon capsid maturation are consistent with previous depletion–reconstitution and dominant-negative studies showing that ABCE1 is important for assembly of Gag and virion production (9). Unfortunately, the post-translational role of ABCE1 in assembly of Gag is not amenable to study using small interfering RNA knockdown of ABCE1 because knockdown of this essential protein leads to rapid loss of translation and cell death as reported by others (11) and in our hands (J.E. Dooher and J.R. Lingappa, unpublished data), necessitating the use of alternate approaches to further document the role of ABCE1 in HIV assembly. It should also be noted that in this kinetic study, we do not address how much intracellular Gag is present in the form of Gag–ABCE1 complexes. This is because all the p24Gag antibodies we have examined to date immunoprecipitate p55Gag polypeptides very inefficiently even under denaturing conditions, thereby preventing us from accurately quantifying Gag–ABCE1 complexes as a percentage of total radiolabeled p55Gag in cells.

Although the exact function of ABCE1 during assembly remains to be determined, the finding that ABCE1 associates transiently with p55Gag during assembly and dissociates at the start of virus maturation is consistent with a chaperone-like mechanism of action for ABCE1. ABCE1 is the sole member of the E family in the ATP-binding cassette superfamily and is highly conserved in eukaryotes and archaea (36). In normal cells, it functions during nucleocytoplasmic transport of ribsosomes and assembly of preinitiation complexes during translation (11–14,21,22). ABCE1 from an archaea was recently crystallized and shown to be capable of undergoing a clamp-like power stroke (37), but the role of this power stroke in transport and assembly of ribosomes or the assembly of primate lentiviral capsids remains to be determined. Notably, these intracellular ABCE1- and Gag-containing assembly intermediates do not correspond to ribosomes or polysomes because they form ∼80, ∼150 and ∼500S complexes even in the presence of EDTA (8,9) (Figure S3), which causes ribosomes to disassemble into their 40 and 60S subunits [e.g. Ramirez et al. (38), Shor et al.(39)]. While the domains in ABCE1 that are important for the Gag–ABCE1 association have not been mapped yet, it is known that basic residues in the NC domain of p55Gag act directly or indirectly to promote Gag–ABCE1 association (7).

Another question that is answered by these studies concerns the location of ABCE1-containing assembly intermediates. Immunogold labeling studies indicate that the partially assembled electron-dense structures that have been recognized as the hallmark of HIV-1 assembly in cells in fact constitute the ABCE1-containing assembly intermediates that have been isolated biochemically from cells. In the past, such assembling Gag structures at the plasma membrane have sometimes been referred to as sites of ‘self-assembly’(40), but our studies suggest that this is a misnomer, given the specific recruitment into these sites of a cellular ATPase ABCE1 that promotes assembly. Here, quantitation of immunogold labeling experiments revealed a specific recruitment of ABCE1 into sites of Gag assembly in cells expressing either wild-type HIV-1 or HIVPro− compared with cells expressing an assembly-defective HIV-1 Gag protein or mock-transfected cells. It should also be noted that some ABCE1 proteins that are colocalized with wild-type Gag clusters at the membrane are likely to have been missed in our micrographs because they were outside the plane of these ∼80-nm thin sections, and because steric hindrance caused by antibodies and gold particles renders labeling of proteins inefficient (41). Interestingly, in virus particles that appeared to have completed assembly, ABCE1 frequently remained in the cytoplasm at the base of the budding virus stalk (seen in numerous particles in Figure 7), consistent with the findings that ABCE1 is not packaged into virions (5) (Figure S2) but rather associates transiently with Gag during assembly, appears to dissociate at the time of maturation (Figure 2) and is left behind in the cell when virions are released. Our finding that ABCE1-containing ∼80/150S assembly intermediates form within minutes after translation (Figures 2 and 5) raises the possibility that ∼80/150S intermediates may be present in the cytoplasm. Cytosolic complexes containing Gag are difficult to identify in our immunogold labeling studies because the small gold particles that are easily seen at the plasma membrane are harder to identify amidst high concentrations of protein that raise the background in the cytoplasm. Thus, biochemical approaches will be required to determine whether ABCE1-containing intermediates are present in the cytoplasm, in addition to the intermediates identified here at the plasma membrane using ultrastructural approaches.

These immunogold EM labeling studies also present the first detailed view of the subcellular localization of primate ABCE1. Previously, immunofluorescence studies revealed that ABCE1 is present in the cytosol and the nucleus in yeast (12–14). It has also been reported to be present in mitochondria (42). Here we confirm the presence of ABCE1 in the cytoplasm, nucleus and nucleolus by immunogold EM labeling, as expected. However, we also demonstrate that ABCE1, which lacks transmembrane domains or motifs for fatty acylation, is recruited into discrete sites at the plasma membrane upon expression of assembly-competent HIV-1 Gag. Additional studies will be required to examine whether specific plasma membrane localization occurs in normal cells and to determine the mechanism underlying recruitment of ABCE1 to the plasma membrane.

Finally, pulse-chase studies presented here also demonstrated that processing of p55Gag by the HIV protease occurs intracellularly and precedes virion release. This had previously been shown in infected CEM cells (10), but here we find similar results using a different HIV-1 protease mutant in transfected COS-1 cells. This previous study had also demonstrated that protease inactivation reduces virus release (10). Here we confirmed that mutational inactivation of the HIV-1 protease led to delayed virion release, and extended these observations by demonstrating that protease inactivation also correlated with prolonged association of p55Gag with ABCE1. Whether delayed dissociation of ABCE1 is a cause or consequence of delayed virus release and how mutational inactivation of the protease leads to delayed dissociation will need to be examined in future studies. Interestingly, we found that immature virus is eventually released from cells expressing HIVPro− and that these released immature virions did not contain ABCE1 (Figure S2). This suggests that dissociation of ABCE1 from p55Gag eventually does occur despite the absence of HIV-1 protease activity, albeit at a much later time. Our finding that protease inactivation correlated both with a prolonged half-life of intracellular Gag–ABCE1 complexes and with reduced virion release raises the possibility that events of capsid assembly, maturation, and virus release are interdependent. Thus, these kinetic studies of intracellular Gag–ABCE1 complexes also demonstrate that assembly is followed in an ordered sequence first by the onset of processing and then by release, and suggest a mechanistic link between maturation and viral–host interactions that occur during assembly.

## Materials and methods

### Plasmids and cell culture

The plasmid for expression of wild-type HIV-1 (HIV) encodes the full-length HIV-1 Bru (LAI) genome with a deletion in the *env* gene and was obtained from Michael Emerman (43). HIVΔNCΔp6 and HIVPro− are identical to the wild-type proviral construct except for the following point mutations that were engineered by site-directed mutagenesis: a double stop codon was inserted after aa 363 of Gag to generate HIVΔNCΔp6, and the protease-inactivating substitutions D25K, G49W and I50W (16) were engineered into the protease gene to generate HIVPro−. All mutations were verified by sequencing. Mock plasmid was pcDNA3.1 (Invitrogen, Carlsbad, CA, USA). COS-1 (ATCC, Manassas, VA, USA) cells were maintained per supplier’s instructions and were transfected in 60-mm dishes using 24 μL Lipofectamine (Invitrogen) and 0.75–3.0 μg plasmid, per manufacturer’s protocol. H9 cells stably expressing the HIV genome were described previously (5).

### Radiolabeling and cell harvests

At ∼36–40 h post-transfection, cells were radiolabeled by incubation in starvation medium (serum-free DME lacking methionine and cysteine) for 30 min, pulsed with 1.5 mL starvation medium containing 250 μCi ^35^S-radiolabeled methionine/cysteine (ICN Pharmaceuticals, Costa Mesa, CA, USA), rinsed with PBS and chased with 2 mL complete DME containing 0.2 mm cysteine and 0.2 mm methionine and 10% FBS. At harvest, medium was collected, and cells were rinsed in PBS. For longer chases, medium was collected at 24 and 48 h into the chase, cells were rinsed with PBS and complete DME was replaced. For harvest under denaturing conditions, cells were scraped in 200 μL 50 mm Tris, 1.0% SDS in the presence of 1:100 protease inhibitor cocktail (Sigma-Aldrich, St Louis, MO, USA), boiled for 5 min and clarified in a microfuge. For preparation of COS-1 cell lysates under native conditions, cells were harvested on ice in 200–300 μL chilled native nonionic buffer (0.625% NP40, 10 mm TrisAc, 50 mm KAc, 100 mm NaCl, pH 7.4) or using the same buffer in the presence of 10 mm EDTA or 1 μg/mL of RNAse A (Qiagen, Valencia, CA, USA) final concentration (incubated for 30 min at 37°C), when noted. Unlabeled H9 cells expressing HIV were harvested in 250 μL chilled native nonionic buffer (0.625% NP40, 10 mm TrisAc, 50 mm KAc, 100 mm NaCl, 4 mm MgAc, pH 7.4) alone or using the same buffer in the presence of 1 mm EDTA or 0.5 U apyrase per microliter. Native harvests were performed in the presence of a protease inhibitor cocktail (Sigma-Aldrich), sheared by 30 passes through the rubber sheath of a 20-gauge angiocath and clarified by centrifugation at 300 × ***g*** for 8 min at 4°C in a GH-3.8 rotor (Allegra 6R centrifuge, Beckman Coulter, Fullerton, CA, USA) and at 18 000 × ***g*** for 45 second in a microfuge. Medium was clarified by centrifugation at 365 × ***g*** for 10 min at 4°C in a GH-3.8 rotor and passed through a 0.22-μm syringe filter. For markers and serial immunoprecipitations, cells were steady-state radiolabeled by a 30-min starve, 1-h pulse and 15-min chase. Virus-like particle production was determined by collecting medium from cells, filtering through a 0.22-μm syringe filter, underlayering medium with 1.0 mL of 30% w/v sucrose in water and centrifuging at 162 500 × ***g*** for 45 min at 4°C in an MLS50 rotor (Beckman Coulter). Preparations of VLP were adjusted to identical volumes, and viral envelopes were disrupted by addition of SDS to a final concentration of 1.0%.

### Immunoprecipitation and western blotting

For markers and all αGag immunoprecipitations, native lysate was denatured with SDS to 1.0%, or for medium to 0.75%, boiled for 5 min and diluted to <0.1% SDS with native harvest buffer (lysate) or 50 mm Tris, 2% BSA, pH 7.4 (media). Immunoprecipitations contained 12.5 μL protein G-agarose beads (Pierce, Rockford, IL, USA) and 2 μL αGag [αHIV-1 p24 monoclonal antibody (mAb) 24-4, AIDS Reference and Reagent Program, D Michael Malim (44,45)]. αABCE1 immunoprecipitations were performed by incubating native lysate with 35–50 μL protein A-TrisAcryl beads (Pierce) coupled to affinity-purified rabbit αABCE1 (9) or nonimmune rabbit IgG. Affinity purification and coupling of ABCE1 antiserum was performed according to the standard protocols (46). Immunoprecipitations in a final volume of ≥500 μL were rotated for 2–4 h at 4°C, washed twice with 0.1 m TrisAc, pH 8.0, 1.0% NP40, 50 mm KAc, 100 mm NaCl and twice with identical buffer lacking NP40. Reactions were eluted in 30–50 μL SDS-PAGE loading buffer, subjected to SDS-PAGE and visualized by autoradiograph or Western blot. For serial immunoprecipitations, parallel αABCE1 immunoprecipitations were eluted by boiling in 200 μL 50 mm Tris, pH 7.4, and 1.0% SDS. Eluates were pooled, divided equally, diluted to <0.1% SDS with 50 mm Tris, 2% BSA, pH 7.4, and subjected to repeat immunoprecipitation with αABCE1 or αGag as described above, or mouse nonimmune antibody [5 μL 5 mg/mL mouse IgG (Sigma-Aldrich) with protein G-agarose or 3 μL αactin (Sigma-Aldrich) with protein A-TrisAcryl].

Western blotting for Gag was performed using primary antibodies αp24Gag (Dako, Carpinteria, CA, USA) or 183-H12-5C (NIH AIDS Reference and Reagent Program, Dr Bruce Chesebro and Kathy Wehrly (47–49)) followed by αmouse IgG_1_ (Santa Cruz Biotechnology, Santa Cruz, CA, USA) as described previously (5).

### Gradient analysis

Native cell lysates (200 μL) from pulse-labeled cells were layered onto 2 mL sucrose-step gradients containing 500 μL each of 20, 40, 50 and 75% sucrose w/v in 0.625% NP40, 10 mm TrisAc, pH 7.4, 50 mm KAc and 100 mm NaCl. Gradients were centrifuged at 134 000 × ***g*** for 45 min at 4°C in a TLS55 rotor (Beckman Coulter) using a Beckman Coulter Optima ultracentrifuge. Fractions (300 μL) were collected serially from the top, subjected to native immunoprecipitation with αABCE1 and analyzed by SDS-PAGE and autoradiography in parallel with Gag and ABCE1 markers. p55Gag band intensity in autoradiographs was determined for each fraction, and values were plotted as relative autoradiography units. Native cell lysates (200 μL) from unlabeled H9 cells were layered onto 2 mL sucrose-step gradients containing 400 μL each of 5, 10, 15 and 30% sucrose w/v in 0.625% NP40, 10 mm TrisAc, pH 7.4, 50 mm KAc, 100 mm NaCl and 4 mm MgAc with or without 10 mm EDTA. Fractions (200 μL) were collected serially from the top and analyzed by SDS-PAGE and Western blotting for Gag. Calibration of gradients with markers for approximate S values has been described previously (8).

### Analysis of western blots and autoradiographs

Autoradiographs and Western blots were digitized using a Duoscan T1200 (Agfa-Gevaert, Ridgefield Park, NJ) or Canoscan 8400F scanner (Canon, Lake Success, NY, USA) and photoshop 7.0 software (Adobe, San Jose, CA, USA). Mean p55Gag and p24Gag band intensities in autoradiographs were determined by quantitating pixels in band aligning with Gag marker and adjusting for band size and background. Ratio of Gag to GagPol was determined by band quantitation as described above, followed by a correction for the number of methionines and cysteines in each band. Quantitation of wild-type HIV versus HIVPro− VLP release was performed as follows: mature wild-type HIV particles were determined by quantitating p55Gag and p24Gag bands, while immature HIVPro− particles (which do not contain any p24Gag) were quantitated using p55Gag. In both cases, p55Gag signal was corrected by multiplying by 0.54 to normalize p55Gag signals to the number of radiolabeled residues present in p24Gag.

### Immunogold labeling

At ∼24 h post-transfection, cells were rinsed with PBS and fixed as indicated below on ice at 4°C for ∼2–5 h. For standard EM, cells were fixed in half-strength Karnovsky’s fixative (2% paraformaldehyde, 2.5% glutaraldehyde in 0.1 m cacodylate buffer). For immunogold EM, cells were fixed in 3% paraformaldehyde and 0.025% glutaraldehyde in 0.1 m phosphate buffer, pH 7.4. Following fixation, cells were scraped, pelleted and washed three times in 0.1 m cacodylate buffer. All cell pellets were dehydrated by successive 15-min treatments of increasing concentrations of ethanol (50, 70, 95, 100%). For standard EM samples, samples were treated repeatedly with propylene oxide and then were infiltrated overnight in 1:2 propylene oxide/Epon resin, and blocks were cured for 48 h at 60°C. Immunogold samples were infiltrated overnight with LR White embedding resin (London Resin Company Ltd, Reading, Berkshire, England, UK) in ethanol, changed to straight LR White, embedded in gelatin capsules (Electron Microscopy Sciences, Hatfield, PA, USA) and cured overnight in a UV light cryochamber at 4°C. Samples were sectioned (∼70 nm), grids treated with 0.05 m glycine for 20 min at room temperature, rinsed in PBS and blocked with bovine serum albumin-C (Electron Microscopy Sciences) in PBS/Tween-20. Primary antibodies αGag [mAb αHIV-1 p24 (Dako)] and αABCE1 that was dialyzed (Slidalyzer, Pierce) and concentrated (Amicon concentrator, Millipore, Billerica, MA, USA) were used with goat αmouse IgG conjugated to 6-nm gold and goat αrabbit IgG conjugated to 15-nm gold (Electron Microscopy Sciences) respectively. For double immunolabeling, grids were incubated with αGag for 1 h at room temperature, then αABCE1 for 1 h, followed by secondary antibodies, fixation with 2% glutaraldehyde in PBS for 10 min at room temperature, with BSA/PBS/Tween-20 rinses between all steps and final rinse in distilled water. Samples were poststained with uranyl acetate for 1 min and lead citrate for 30 seconds. Similar immunolabeling was obtained using secondary antibodies conjugated to 6- and 15-nm gold particles, for Gag and ABCE1 labeling, respectively. All samples were visualized with a JEOL 1010 or JEOL 1230 microscope (JEOL, Peabody, MA, USA). Cell pellet processing, immunolabeling and EM were performed by the Fred Hutchinson EM Core Facility (Seattle, WA, USA). Negatives of photomicrographs were scanned, and for preparation of figures, the digital images were adjusted in Adobe Photoshop (Adobe) using the curves function applied uniformly to the entire image in order to best visualize electron-dense gold particles.

### Quantitation of immunolabeling

In comparing cells expressing wild-type HIV with cells expressing HIVΔNCΔp6, an experienced microscopist provided printed nondigital photomicrographs of random fields depicting wild-type Gag and GagΔNCΔp6 labeling near the plasma membrane. Colocalization was determined by counting ABCE1 labeling within 100 nm of Gag clusters in these photomicrographs, the approximate diameter of immature HIV-1 capsids. A cluster of Gag was defined as a group of two or more Gag labeling events within 100 nm of the plasma membrane. To avoid counting clusters twice, we defined a minimum separation distance (100 nm) between clusters. Percentage of colocalization was defined as the number of Gag clusters with ABCE1 within 100 nm of Gag, as a percentage of total clusters. Error bars represent standard error of the mean (SEM) from three separate experiments, with a total number of 172 and 372 wild-type Gag and GagΔNCΔp6 clusters, counted from 64 and 44 fields, respectively.

In comparing cells expressing HIVPro− with mock-transfected cells, an experienced microscopist randomly selected 6 HIVPro− cells that exhibited virus production and then photographed randomly selected areas of plasma membrane in these cells that contained budding virus particles, producing 24 digital micrographs. Randomly selected areas of plasma membrane from five randomly selected mock-transfected cells were also photographed to produce 17 digital micrographs. In the HIVPro− cells, only areas of membrane with visible budding virus particles were analyzed (including a 100-nm margin on either side of the budding virus cluster), while in mock cells, all plasma membrane regions seen in the micrographs were scored. ABCE1 labeling was scored as positive only when it was within 100 nm of plasma membrane that was analyzed. For both groups, the length of membrane on each digital micrograph was determined using a curve perimeter tool in Canvas X (Victoria, BC, Canada) and the amount of ABCE1 labeling in the selected region was determined.

## Supplementary Materials

Figure S1 HIV Gag is associated with ABCE1 independently of ribosomes or polysomes. COS-1 cells expressing HIV (lanes 3–5) or mock plasmid (lanes 6–8) were pulse radiolabeled with 35S-methionine/cysteine and chased with unlabeled media. Lysates were harvested under native conditions using (A) buffer lacking EDTA (lanes 3 and 6), (B) buffer containing 10mM EDTA (lanes 4 and 7) or (C) buffer containing RNAse A but no EDTA (lanes 5 and 8), and were immunoprecipitated with αABCE1. Markers (M) for the position of Gag and ABCE1 are shown. Dot indicates position of coimmunoprecipitated p55Gag found in lanes 3–5. Molecular weight markers are shown to the right.

Figure S2 ABCE1 is not present in substantial quantities in virions released from cells expressing HIVPro2. A) COS-1 cells were transfected to express HIV, HIVPro-, HIVδNCδp6 or mock plasmid, as shown by Western blotting (WB) aliquots of cell lysate with aGag. B) Cell lysates were subjected to immunoprecipitation with aABCE1 under native conditions followed by Western blotting with either αABCE1 (lanes 5–8, top panel) or αGag (lanes 5–8, bottom panel) to show the relative ratio of ABCE1 and Gag present in intracellular complexes. VLPs were collected by sedimenting medium from these cells through a sucrose cushion. ABCE1 was not detected in VLP preparations (lanes 9–12), which contained amounts of Gag equal to or greater than those found in the immunoprecipitated intracellular complexes. Immunoprecipitation of VLP aliquots with αABCE1 also failed to reveal ABCE1 in VLPs (data not shown). All lanes in panel B were analyzed in parallel, with the dividing line indicating splicing of lanes from a single film. Molecular weight markers are shown to the left in all panels. HC indicates IgG heavy chain detected in immunoprecipitations.

Figure S3 Chelation of divalent cations mimics effect of ATP depletion in enhancing Gag present in ∼80/150S complexes. Human H9 cells stably expressing the HIV-1 provirus were harvested in nondetergent buffer containing Mg^2+^, Mg^2+^ and apyrase (which depletes ATP), or EDTA, and analyzed by velocity sedimentation and Western blotting for Gag. Positions of ∼10S and ∼80/150S complexes are indicated. Data are representative of more than four experiments.

Host ABCE1 is at plasma membrane HIV assembly sites and its dissociation from Gag is linked to subsequent events of virus production

Julia E. Dooher, Bobbie L. Schneider, Jonathan C. Reed, and Jaisri R. Lingappa

The supplemental materials for this materials is available through the Traffic home page at: http://www.traffic.dk/suppmat/8_3b.asp

These supplemental data are provided by the authors, and hosted on the Traffic home page. Blackwell-Munksgaard maintains and operates this site.

## References

[1] Briggs JA, Simon MN, Gross I, Krausslich HG, Fuller SD, Vogt VM, Johnson MC (2004). The stoichiometry of Gag protein in HIV-1. Nat Struct Mol Biol.

[2] Campbell S, Fisher RJ, Towler EM, Fox S, Issaq HJ, Wolfe T, Phillips LR, Rein A (2001). Modulation of HIV-like particle assembly in vitro by inositol phosphates. Proc Natl Acad Sci USA.

[3] Campbell S, Rein A (1999). In vitro assembly properties of human immunodeficiency virus type 1 Gag protein lacking the p6 domain. J Virol.

[4] Campbell S, Vogt VM (1997). In vitro assembly of virus-like particles with Rous sarcoma virus Gag deletion mutants: identification of the p10 domain as a morphological determinant in the formation of spherical particles. J Virol.

[5] Dooher JE, Lingappa JR (2004). Conservation of a stepwise, energy-sensitive pathway involving HP68 for assembly of primate lentivirus capsids in cells. J Virol.

[6] Dooher JE, Lingappa JR (2004). Cell-free systems for capsid assembly of primate lentiviruses from three different lineages. J Med Primatol.

[7] Lingappa JR, Dooher JE, Newman MA, Kiser PK, Klein KC (2006). Basic residues in the nucleocapsid domain of Gag are required for interaction of HIV-1 gag with ABCE1 (HP68), a cellular protein important for HIV-1 capsid assembly. J Biol Chem.

[8] Lingappa JR, Hill RL, Wong ML, Hegde RS (1997). A multistep, ATP-dependent pathway for assembly of human immunodeficiency virus capsids in a cell-free system. J Cell Biol.

[9] Zimmerman C, Klein KC, Kiser PK, Singh ARS, Firestein BL, Riba SC, Lingappa JR (2002). Identification of a host protein essential for assembly of immature HIV-1 capsids. Nature.

[10] Kaplan AH, Manchester M, Swanstrom R (1994). The activity of the protease of human immunodeficiency virus type 1 is initiated at the membrane of infected cells before the release of viral proteins and is required for release to occur with maximum efficiency. J Virol.

[11] Chen ZQ, Dong J, Ishimura A, Daar I, Hinnebusch AG, Dean M (2006). The essential vertebrate ABCE1 protein interacts with eukaryotic initiation factors. J Biol Chem.

[12] Dong J, Lai R, Nielsen K, Fekete CA, Qiu H, Hinnebusch AG (2004). The essential ATP-binding cassette protein RLI1 functions in translation by promoting preinitiation complex assembly. J Biol Chem.

[13] Kispal G, Sipos K, Lange H, Fekete Z, Bedekovics T, Janaky T, Bassler J, Aguilar Netz DJ, Balk J, Rotte C, Lill R (2005). Biogenesis of cytosolic ribosomes requires the essential iron-sulphur protein Rli1p and mitochondria. EMBO J.

[14] Yarunin A, Panse VG, Petfalski E, Dez C, Tollervey D, Hurt EC (2005). Functional link between ribosome formation and biogenesis of iron-sulfur proteins. EMBO J.

[15] Singh AR, Hill RL, Lingappa JR (2001). Effect of mutations in Gag on assembly of immature human immunodeficiency virus type 1 capsids in a cell-free system. Virology.

[16] McPhee F, Good AC, Kuntz ID, Craik CS (1996). Engineering human immunodeficiency virus 1 protease heterodimers as macromolecular inhibitors of viral maturation. Proc Natl Acad Sci USA.

[17] Jacks T, Power MD, Masiarz FR, Luciw PA, Barr PJ, Varmus HE (1988). Characterization of ribosomal frameshifting in HIV-1 gag-pol expression. Nature.

[18] Wilson W, Braddock M, Adams SE, Rathjen PD, Kingsman SM, Kingsman AJ (1988). HIV expression strategies: ribosomal frameshifting is directed by a short sequence in both mammalian and yeast systems. Cell.

[19] Wills JW, Craven RC (1991). Form, function, and use of retroviral gag proteins. AIDS.

[20] Tobin GJ, Nagashima K, Gonda MA (1996). Immunologic and ultrastructural characterization of HIV pseudovirions containing Gag and Env precursor proteins engineered in insect cells. Methods.

[21] Estevez AM, Haile S, Steinbuchel M, Quijada L, Clayton C (2004). Effects of depletion and overexpression of the Trypanosoma brucei ribonuclease L inhibitor homologue. Mol Biochem Parasitol.

[22] Zhao Z, Fang LL, Johnsen R, Baillie DL (2004). ATP-binding cassette protein E is involved in gene transcription and translation in Caenorhabditis elegans. Biochem Biophys Res Commun.

[23] Gelderblom HR, Hausmann EH, Ozel M, Pauli G, Koch MA (1987). Fine structure of human immunodeficiency virus (HIV) and immunolocalization of structural proteins. Virology.

[24] Mergener K, Facke M, Welker R, Brinkmann V, Gelderblom HR, Krausslich HG (1992). Analysis of HIV particle formation using transient expression of subviral constructs in mammalian cells. Virology.

[25] Schatzl H, Gelderblom HR, Nitschko H, von der Helm K (1991). Analysis of non-infectious HIV particles produced in presence of HIV proteinase inhibitor. Arch Virol.

[26] Chatel-Chaix L, Clement JF, Martel C, Beriault V, Gatignol A, DesGroseillers L, Mouland AJ (2004). Identification of Staufen in the human immunodeficiency virus type 1 Gag ribonucleoprotein complex and a role in generating infectious viral particles. Mol Cell Biol.

[27] Ding L, Derdowski A, Wang JJ, Spearman P (2003). Independent segregation of human immunodeficiency virus type 1 Gag protein complexes and lipid rafts. J Virol.

[28] Halwani R, Khorchid A, Cen S, Kleiman L (2003). Rapid localization of Gag/GagPol complexes to detergent-resistant membrane during the assembly of human immunodeficiency virus type 1. J Virol.

[29] Lee YM, Liu B, Yu XF (1999). Formation of virus assembly intermediate complexes in the cytoplasm by wild-type and assembly-defective mutant human immunodeficiency virus type 1 and their association with membranes. J Virol.

[30] Lee YM, Yu XF (1998). Identification and characterization of virus assembly intermediate complexes in HIV-1-infected CD4+ T cells. Virology.

[31] Morikawa Y, Goto T, Momose F (2004). Human immunodeficiency virus type 1 Gag assembly through assembly intermediates. J Biol Chem.

[32] Simon JH, Carpenter EA, Fouchier RA, Malim MH (1999). Vif and the p55(Gag) polyprotein of human immunodeficiency virus type 1 are present in colocalizing membrane-free cytoplasmic complexes. J Virol.

[33] Tritel M, Resh MD (2000). Kinetic analysis of human immunodeficiency virus type 1 assembly reveals the presence of sequential intermediates. J Virol.

[34] Tritel M, Resh MD (2001). The late stage of human immunodeficiency virus type 1 assembly is an energy-dependent process. J Virol.

[35] Dooher JE, Pineda MJ, Overbaugh J, Lingappa JR (2004). Characterization of virus infectivity and cell-free capsid assembly of SIVMneCL8. J Med Primatol.

[36] Kerr ID (2004). Sequence analysis of twin ATP binding cassette proteins involved in translational control, antibiotic resistance, and ribonuclease L inhibition. Biochem Biophys Res Commun.

[37] Karcher A, Buttner K, Martens B, Jansen RP, Hopfner KP (2005). X-ray structure of RLI, an essential twin cassette ABC ATPase involved in ribosome biogenesis and HIV capsid assembly. Structure (Camb).

[38] Ramirez M, Wek RC, Hinnebusch AG (1991). Ribosome association of GCN2 protein kinase, a translational activator of the GCN4 gene of Saccharomyces cerevisiae. Mol Cell Biol.

[39] Shor B, Calaycay J, Rushbrook J, McLeod M (2003). Cpc2/RACK1 is a ribosome-associated protein that promotes efficient translation in Schizosaccharomyces pombe. J Biol Chem.

[40] Nermut MV, Hockley DJ (1996). Comparative morphology and structural classification of retroviruses. Curr Top Microbiol Immunol.

[41] Hermann R, Walther P, Muller M (1996). Immunogold labeling in scanning electron microscopy. Histochem Cell Biol.

[42] Le Roy F, Bisbal C, Silhol M, Martinand C, Lebleu B, Salehzada T (2001). The 2-5A/RNase L/RNase L inhibitor (RNI) pathway regulates mitochondrial mRNAs stability in interferon alpha-treated H9 Cells. J Biol Chem.

[43] Kimpton J, Emerman M (1992). Detection of replication-competent and pseudotyped human immunodeficiency virus with a sensitive cell line on the basis of activation of an integrated beta-galactosidase gene. J Virol.

[44] Fouchier RA, Meyer BE, Simon JH, Fischer U, Malim MH (1997). HIV-1 infection of non-dividing cells: evidence that the amino-terminal basic region of the viral matrix protein is important for Gag processing but not for post-entry nuclear import. EMBO J.

[45] Simon JH, Fouchier RA, Southerling TE, Guerra CB, Grant CK, Malim MH (1997). The Vif and Gag proteins of human immunodeficiency virus type 1 colocalize in infected human T cells. J Virol.

[46] Harlow E, Lane D (1999). Using Antibodies. A Laboratory Manual.

[47] Chesebro B, Wehrly K, Nishio J, Perryman S (1992). Macrophage-tropic human immunodeficiency virus isolates from different patients exhibit unusual V3 envelope sequence homogeneity in comparison with T-cell-tropic isolates: definition of critical amino acids involved in cell tropism. J Virol.

[48] Toohey K, Wehrly K, Nishio J, Perryman S, Chesebro B (1995). Human immunodeficiency virus envelope V1 and V2 regions influence replication efficiency in macrophages by affecting virus spread. Virology.

[49] Wehrly K (1997). Chesebro B. p24 antigen capture assay for quantification of human immunodeficiency virus using readily available inexpensive reagents. Methods.

