# Supplementary figures and images for: Host ABCE1 is at Plasma Membrane HIV Assembly Sites and Its Dissociation from Gag is Linked to Subsequent Events of Virus Production
Source: Traffic. 2007 Jan 15;8(3):195–211. doi: 10.1111/j.1600-0854.2006.00524.x (PMC1865004; doi:10.1111/j.1600-0854.2006.00524.x)

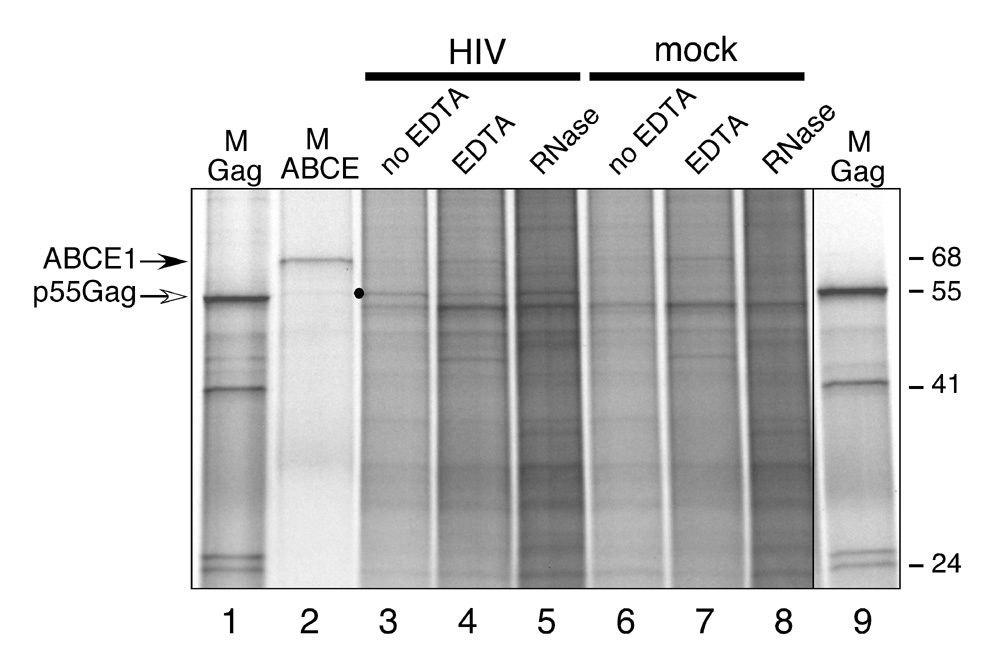

Supplement: Figure S1 — HIV Gag is associated with ABCE1 independently of ribosomes or polysomes. [file tra0008-0195_8_3bfig1.jpg]

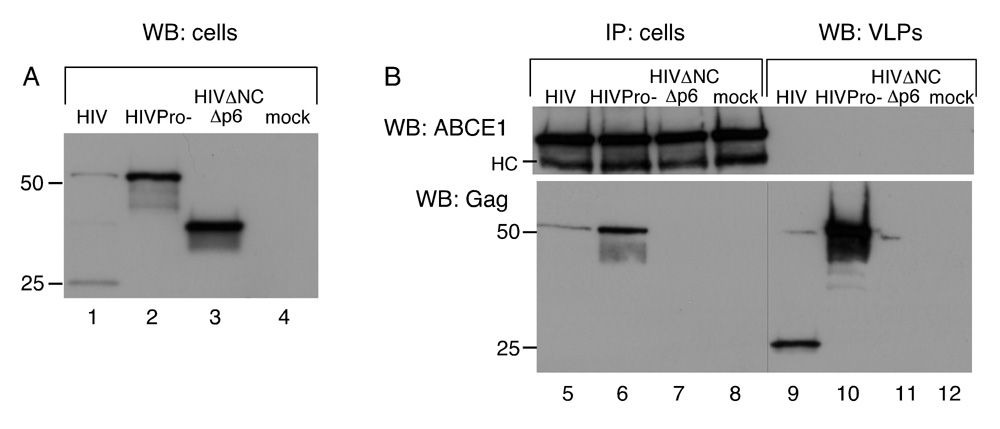

Supplement: Figure S2 — ABCE1 is not present in substantial quantities in virions released from cells expressing HIVPro2. [file tra0008-0195_8_3bfig2.jpg]

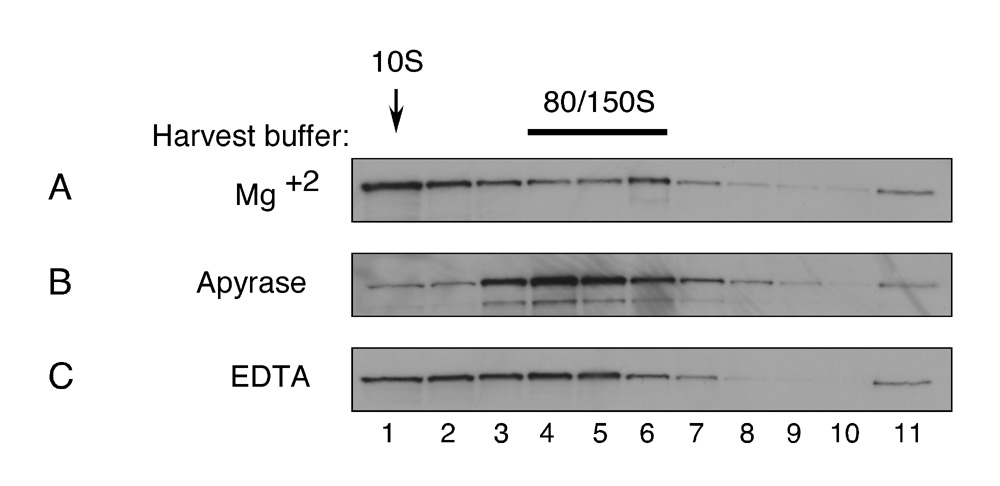

Supplement: Figure S3 — Chelation of divalent cations mimics effect of ATP depletion in enhancing Gag present in ∼80/150S complexes. [file tra0008-0195_8_3bfig3.jpg]
